# Supplementary material for: Fabrication of Sulfated Heterosaccharide/Poly (Vinyl Alcohol) Hydrogel Nanocomposite for Application as Wound Healing Dressing
Source: Molecules. 2022 Mar 10;27(6):1801. doi: 10.3390/molecules27061801 (PMC8955895; doi:10.3390/molecules27061801)
Supplement: Supplementary file 1 [file molecules-27-01801-s001.zip › molecules-1568117-supplementary.pdf]

Supporting Information

Fabrication of sulfated heterosaccharide/poly (vinyl alcohol) hydrogel nanocomposite for application as wound healing dressing

Yang Liu[1,2], Ning Wu[2], Lihua Geng[2,3], Yang Yue[2,3], Quanbin Zhang[2,3], Jing Wang[2,3*]

[1.] Qing Dao University, 308 Ningxia Road, Qingdao 266003, PR China;

[2.] CAS and Shandong Province Key Laboratory of Experimental Marine Biology, Center for Ocean Mega-Science, Institute of Oceanology, Chinese Academy of Sciences, 7 Nanhai Road, Qingdao, 266071, PR China;

[3.] Laboratory for Marine Biology and Biotechnology, Qingdao National Laboratory for Marine Science and Technology, Wenhai Road, Aoshanwei, Jimo, Qingdao 266237, China;

1-Tensile test of PVA hydrogel and sulfated heterosaccharide/poly(vinyl alcohol) hydrogel nanocomposite

Cut the hydrogel into 1 cm × 3 cm strips. Measure their thickness and record. Four parallel samples of each hydrogel were tested in a universal material testing machine (MTS Systems Corp., LSB.203 D) at a speed of 10 mm/min.

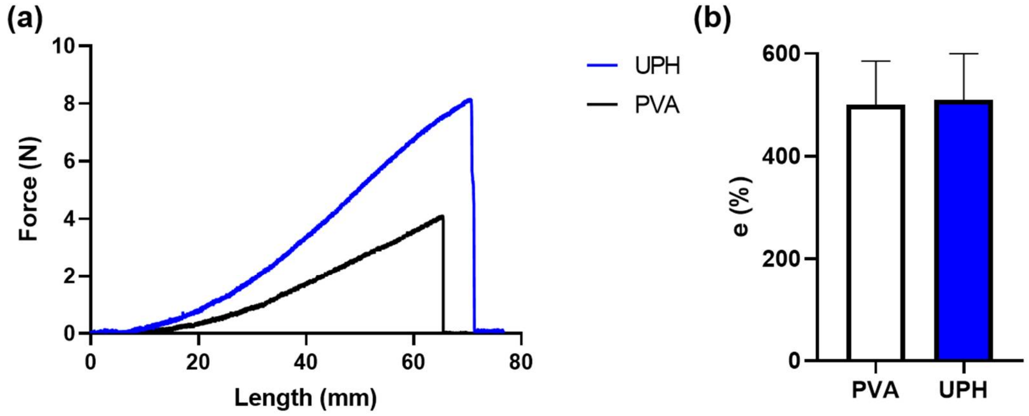

Figure S1. (a) UPH and PVA stress-strain curves; (b) percentage of breaking elongation of UPH and PVA

As shown in Figure S1, PVA hydrogels and UPH had approximately 500% of breaking elongation, and their tensile curves basically conform to the characteristics of elastomers.
